# Primary nonfunction following liver transplantation: Learning of graft metabolites and building a predictive model

**DOI:** 10.1002/ctm2.483

**Published:** 2021-07-08

**Authors:** Xueyou Zhang, Cheng Zhang, Haitao Huang, Ruihan Chen, Yimou Lin, Leiming Chen, Lili Shao, Jimin Liu, Qi Ling

**Affiliations:** ^1^ Department of Surgery The First Affiliated Hospital Zhejiang University School of Medicine Hangzhou China; ^2^ Key Lab of Combined Multi‐Organ Transplantation Ministry of Public Health Hangzhou China; ^3^ Health Management Center the First Affiliated Hospital Zhejiang University School of Medicine Hangzhou China; ^4^ Department of Pathology and Molecular Medicine Faculty of Health Sciences McMaster University Hamilton Canada

Dear Editor,

Primary nonfunction (PNF) is defined as the need for emergent re‐transplant when a graft never presented any evidence of initial function following liver transplantation (LT) after excluding other causes such as acute cellular rejection or hepatic artery thrombosis.^1^ The cause of PNF is believed to be associated with graft quality, but the mechanism is largely unknown.^1^, ^2^, ^3^ With the increasing demand for extended criteria donors due to organ shortage, the precise assessment of graft quality, prediction, and early prevention of PNF become a major challenge.^2^, ^4^ This letter was written to present the first pilot‐scale study, which determined the grafts’ metabolic profiling of developing PNF and constructed an integrated graft metabolites and clinical parameters‐based PNF (GMCP‐PNF) predictive model.

We included 399 adult patients who underwent primary LT from donation after citizens’ death between January 2015 and December 2017 in our center (Figure S1). This study complies with the guidelines of the China Ethical Committee and the declaration of Helsinki. Organs from executed prisoners were not used. Informed consents were obtained. Patient characteristics are listed in Table S1. PNF occurred in 14 (3.5%) patients. We analyzed clinical parameters (donor, recipient, and surgical procedure) and found significant risk factors for developing PNF using univariate logistic analysis (Table 1). In multivariate analysis, donor total bilirubin (TB) > 2 ng/ml, graft weight > 1.5 kg, cold ischemia time (CIT) > 10 h, and graft warm ischemia time (GWIT) > 60 min were discovered as independent risk factors of PNF (Table 1). No recipient parameters were found to be independent risk factors of PNF.

**TABLE 1:** The potential risk factors of primary nonfunction

	Univariate		Multivariate^a^	
	OR (95%, CI)	*p‐*value	OR (95%, CI)	*p‐*value
Quantitative data				
Donor TB	1.019 (0.997, 1.041)	0.087		
Donor AST	1.003 (1.000, 1.007)	0.045		
Donor ALT	1.002 (1.000, 1.004)	0.065		
Graft weight	1.001 (1.000, 1.003)	0.086		
CIT	1.260 (1.100, 1.444)	0.001		
GWIT	1.059 (1.025, 1.095)	0.001		
Anhepatic time	1.037 (1.018, 1.056)	0.002		
MELD score	1.052 (1.006, 1.100)	0.027		
Categorical data^b^				
Donor TB > 2 ng/ml	4.443 (1.394, 14.16)	0.012	7.488 (1.834, 30.57)	0.005
Donor AST > 120 U/L	4.065 (1.245, 13.27)	0.020		
Donor ALT > 40 U/L	3.460 (1.023, 11.70)	0.046		
Graft weight > 1.5 kg	3.755 (1.271, 11.09)	0.017	4.448 (1.216, 16.28)	0.024
CIT > 10 h	7.054 (2.321, 21.44)	0.001	10.67 (2.547, 44.66)	0.001
GWIT > 60 min	5.267 (1.716, 16.17)	0.004	6.858 (1.885, 24.95)	0.003
Anhepatic time > 80 min	4.738 (1.552, 14.46)	0.006		
MELD score > 25	5.047 (1.386, 18.38)	0.014		

Abbreviations: ALT, alanine aminotransferase; AST, aspartate aminotransferase; CI, confidence interval; CIT, cold ischemia time; GWIT, graft warm ischemia time; MELD, model for end‐stage liver diseases; OR, odds ratio; TB, total bilirubin.

^a^ Only categorical data showing significance in univariate analysis were entered into multivariate analysis.

^b^ Cut‐off values were selected according to the ROC curve considered both sensitivity and specificity.

Compared to PNF, early allograft dysfunction (EAD)^5^ is a less severe form of poor graft function immediately after LT, which occurs more often and is associated with lower mortality.^4^ In this study, 35.0% (128/366) of patients developed EAD and had significantly reduced survival as compared to those without EAD (Figure S2). In multivariate analysis, the clinical risk factors for developing EAD and PNF remained the same as well as different (Table S2).

Previous studies have shown the value of metabolomics in the evaluation of EAD.^6^, ^7^ To identify the specific molecular features of PNF and EAD, we performed untargeted metabolomics on fresh liver graft tissues before implantation. The samples were classified into three groups according to the outcomes as PNF group (*n* = 14), the EAD group (*n* = 24), and the control group (*n* = 43). Samples in EAD and control groups were randomly selected based on power calculation (>0.8).^8^ Graft characteristics are shown in Table S3. Partial least‐squares discriminant analysis (PLS‐DA) showed a distinct separation between the PNF and control group on score plots, while the EAD group was positioned between them (Figure 1A). Compared with the control group, the PNF and EAD groups showed 57 and 74 significantly differentially expressed metabolic features (*p* < 0.05). The top 25 metabolites are shown in Figure 1B. The PNF‐associated metabolites were enriched in beta‐oxidation of very‐long‐chain fatty acids, alpha‐linolenic acid, and linoleic acid metabolism, etc. (Figure 1C). The EAD‐associated metabolites were enriched in beta‐oxidation of very‐long‐chain fatty acids, thiamine metabolism, pyruvate metabolism, etc. (Figure 1D). We overlapped the enriched pathways and observed that both PNF‐ and EAD‐associated metabolites were enriched in 24 common pathways, including fatty acid, alanine, aspartate, thiamine, and riboflavin metabolism, urea cycle, and ammonia recycling (Figure 1E). The representative metabolites, which were enriched in the common pathways, are shown in Figure 1F.

**FIGURE 1:**
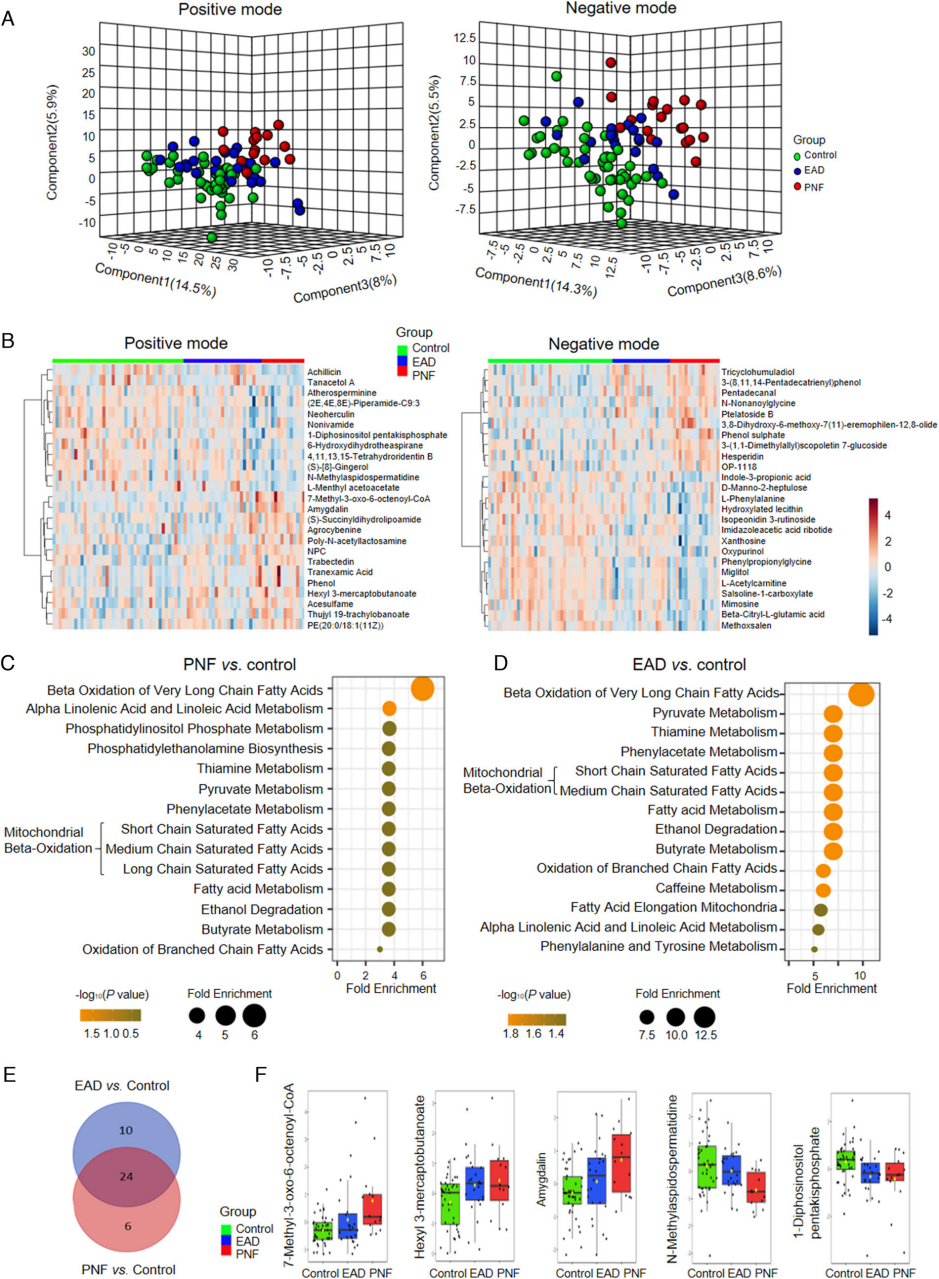
Graft metabolomic features of primary nonfunction (PNF) and early allograft dysfunction (EAD) using UPLC‐MS. (A) Partial least‐squares discriminant analysis (PLS‐DA) score plots in both ESI^+^ and ESI^‐^ models, (B) heatmap showing the clustering result for top 25 metabolites between the three groups with variable importance in projection (VIP) > 1, (C) metabolic pathways undergoing significant changes during PNF, (D) metabolic pathways undergoing significant changes during EAD, (E) the overlapped pathways, (F) the representative metabolites, which were enriched in the common pathways

To further determine the difference between PNF and EAD, we directly compared the PNF and EAD groups and found significant segregation as shown by PLS‐DA score plots (Figure 2A). There were 59 significantly differentially expressed metabolic features between the two groups (*p* < 0.05; Figure 2B). Out of the 59 metabolic features, 21 and six were overlapped with those significantly differentially expressed between the PNF and control groups and between the EAD and control groups, respectively. In contrast, more than half (32/59) were new features discriminating between the PNF and EAD group, indicating potential distinct in the disease etiology. The metabolites were enriched in tryptophan metabolism, pyrimidine metabolism, etc. (Figure 2C).

**FIGURE 2:**
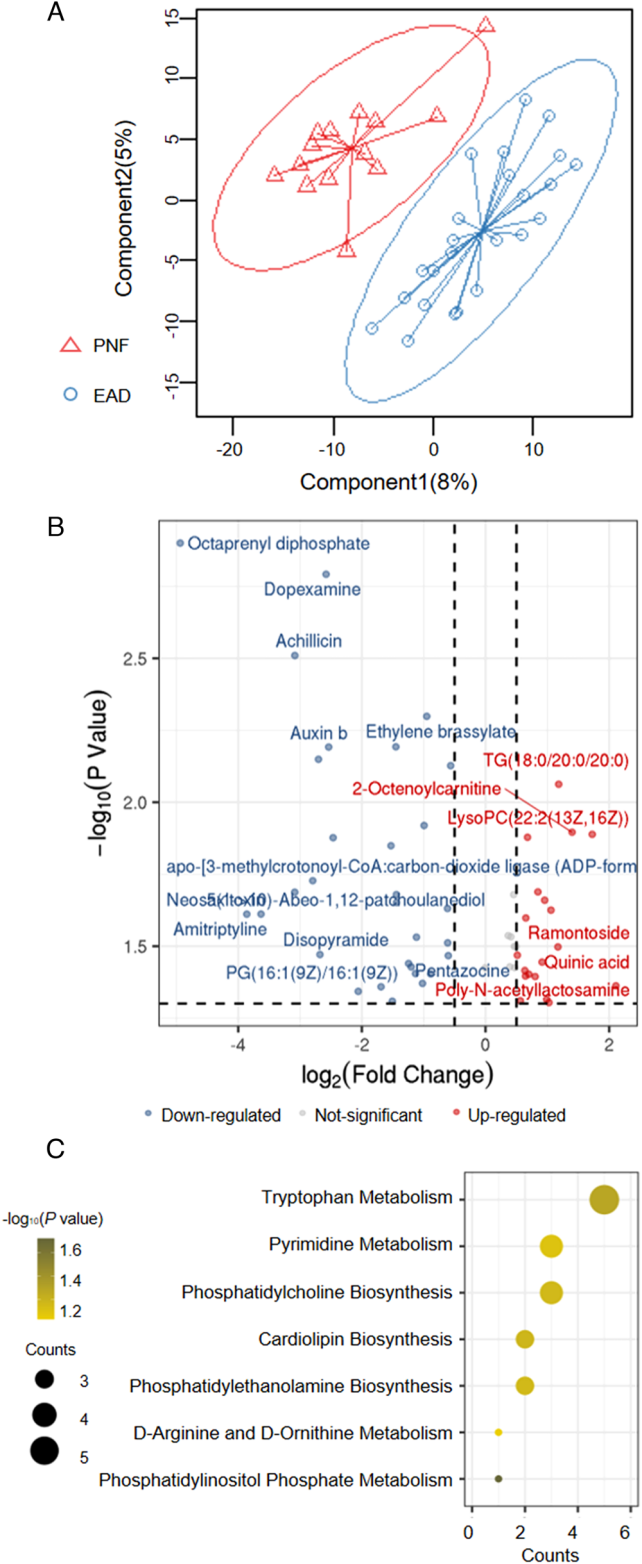
The comparison of graft metabolic features between primary nonfunction (PNF) and early allograft dysfunction (EAD). (A) partial least‐squares discriminant analysis (PLS‐DA) score plots of the two groups, (B) the volcano plot showing the differential expressed metabolic features in both ESI^+^ and ESI^‐^ models, (C) the metabolite set enrichment analysis of PNF groups compared with EAD groups

To finally distinguish organs with a high risk of developing PNF from the others, we compared the PNF group (*n* = 14) to the non‐PNF group (*n* = 67) in their metabolic profiles. The flowchart of the methodology is shown in Figure 3A. First, we performed the least absolute shrinkage and selection operator (Lasso)^9^ to select the key PNF‐specific metabolic features. We used cross‐validation, feature selection, and regularization to prevent overfitting. We obtained PNF‐specific metabolic profiling with eight key metabolites, including achillicin, 3‐hydroxypropanal (HPA), LysoPC(22:2(13Z,16Z)), 3‐oxododecanoic acid glycerides, dopexamine, and 7‐methyl‐3‐oxo‐6‐octenoyl‐CoA (Figure 3B). The eight metabolites were not significantly correlated with graft clinical parameters, indicating their independence (Figure 3C). Second, we performed principal component analysis and extracted the eight metabolites as a virtual super‐biomarker (Figure 3D), which displayed an area under curve (AUC) of 0.930 in predicting PNF. At last, we combined the virtual super‐biomarker with the clinical parameters using logistic regression to construct a GMCP‐PNF predictive model, which showed excellent diagnostic ability (Figure 3E). The model was further tested with the leave‐one‐out cross‐validation (Figure 3F).^10^ Notably, out of three cases predicted as PNF in the EAD group by the model, two suffered early death due to graft failure. Therefore, understanding the metabolic profiling could help clarify the disease pathophysiology and distinguish the more severe type of EAD suffering early death from the other EAD.

**FIGURE 3:**
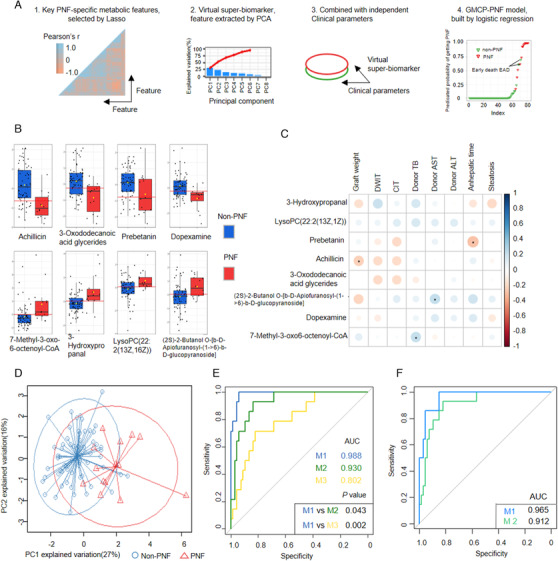
The construction of integrated models for predicting primary nonfunction (PNF). (A) flowchart for model construction, (B) the eight key metabolites based on feature selection and represented the PNF‐specific metabolic profiling, (C) the correlation between eight metabolites and graft clinical parameters, (D) feature extract with principal component analysis (PCA) based on feature‐selected metabolites, (E) the logistic regression model based on graft clinical parameters and extracted metabolomic features could accurately identify PNF. M1: an integrated model, model based on graft clinical parameters and extracted metabolomic feature with the area under curves (AUC) of 0.988, the accuracy of 0.951, specificity of 0.940, and sensitivity of 1.000. M2: metabolites model, model based on an extracted metabolomic feature only, with AUC of 0.930, the accuracy of 0.852, specificity of 0.836, and sensitivity of 0.929. M3: clinical model, model based on graft clinical parameters only. (F) The model was further validated by leave‐one‐out cross‐validation, a resampling technology. M1: the integrated graft metabolites and clinical parameters‐based PNF (GMCP‐PNF), AUC of 0.965, the accuracy of 0.877, specificity of 0.851, and sensitivity of 1.000. M2: the virtual super‐biomarker, AUC of 0.912, the accuracy of 0.840, specificity of 0.821, and sensitivity of 0.929. *p‐value < 0.05

In summary, it was a pioneering study to investigate the grafts’ metabolic profiling of PNF and make a distinction of that with EAD. There was not only similarity but also diversity in the metabolic features between grafts developing PNF and EAD. Furthermore, this study established an integrated GMCP‐PNF predictive model that presented excellent diagnostic value. The work shed light on the deep understanding and clinical use of graft metabolomics profiling, which could be a powerful tool for evaluating graft quality and predicting clinical outcomes if the time for sample processing and analysis could be significantly shortened.

## CONFLICT OF INTEREST

The authors declare no conflict of interest.

## ETHICS APPROVAL

The study was approved by the Ethics Committee of the First Affiliated Hospital, Zhejiang University School of Medicine, China, and following the declaration of Helsinki. Informed consents were obtained from all donors and the families.

## FUNDING INFORMATION

National Natural Science Foundation of China, Grant Numbers: 81771713 and 82011530442; Fundamental Research Funds for the Central Universities, Grant Number: 2019QNA7030.

## AUTHOR CONTRIBUTIONS

Conceptualization: Qi Ling; methodology: Xueyou Zhang; formal analysis: Xueyou Zhang, Cheng Zhang, and Haitao Huang; investigation: Ruihan Chen and Yimou Lin; data curation: Leiming Chen and Lili Shao; writing‐original draft preparation: Xueyou Zhang and Cheng Zhang; and writing‐review and editing: Jimin Liu and Qi Ling.

## DATA AVAILABILITY STATEMENT

The data used in this study can be obtained upon request from corresponding author.

## Supporting information


**Figure S1** Flowchart of the enrollment of patients
**Figure S2** Patient cumulative survival comparison between early allograft dysfunction (EAD) group and non‐EAD group
**Table S1** Patients characteristics
**Table S2** The risk factors of early allograft dysfunction
**Table S3** Graft characteristics in metabolomic analysis
