# Supplementary material for: Primary nonfunction following liver transplantation: Learning of graft metabolites and building a predictive model
Source: Clin Transl Med. 2021 Jul 8;11(7):e483. doi: 10.1002/ctm2.483 (PMC8265168; doi:10.1002/ctm2.483)
Supplement: Supplementary file 1 — Figure S1 Flowchart of the enrollment of patients Figure S2 Patient cumulative survival comparison between early allograft dysfunction (EAD) group and non‐EAD group Table S1 Patients characteristics Table S2 The risk factors of early allograft dysfunction Table S3 Graft characteristics in metabolomic analysis [file CTM2-11-e483-s001.docx]

**FIGURES**

Figure S1 Flowchart of the enrollment of patients.


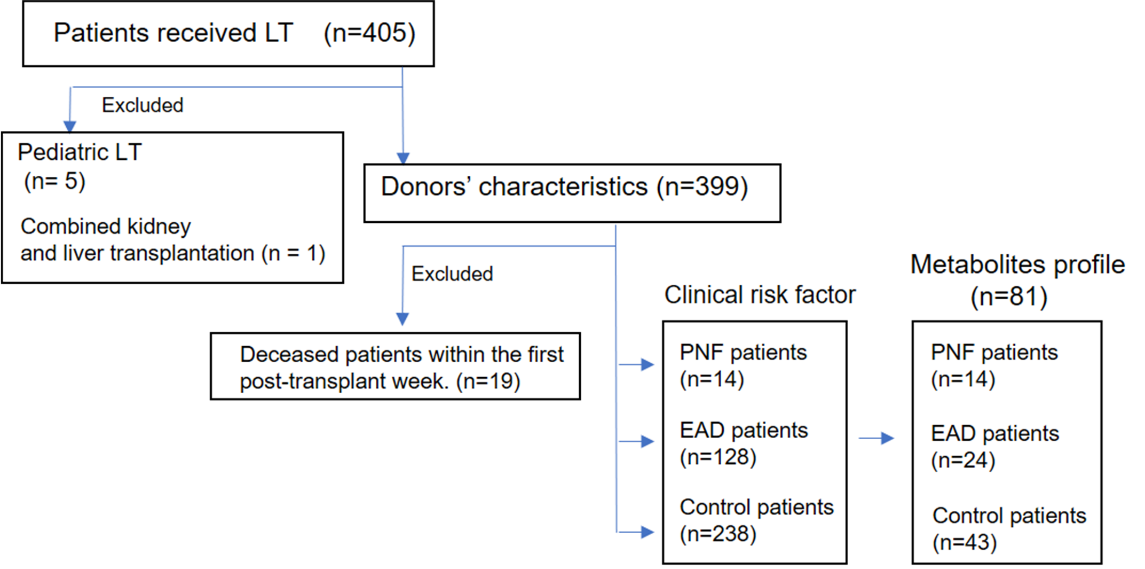


Figure S2 Patient cumulative survival comparison.

**
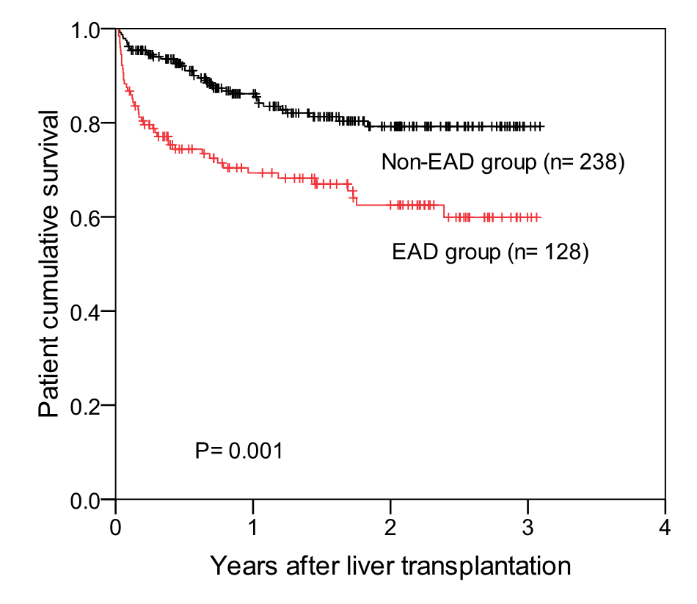
**

Patient cumulative survival comparison between the early allograft dysfunction (EAD) group and the non-EAD group.

**TABLES**

Table S1 Patients characteristics

| Donor characteristic |  |
| --- | --- |
| Age (y) | 42.2 ± 13.7 |
| Male (n/%) | 337 (84.4) |
| BMI (kg/m^2^) | 22.8 ± 2.7 |
| ABO Incompatible | 78 (19.5) |
| DCD (n/%) | 299 (74.9) |
| Cause of death |  |
| Trauma | 245 (61.4) |
| CVA | 139 (34.8) |
| Other | 15 (3.8) |
| HBsAg positive (n/%) | 45 (11.3) |
| Graft weight (kg) | 1.40 ± 0.29 |
| Macrovesicular steatosis (n/%) | 26 (6.5) |
| DWIT (min) | 10.0 (4.0, 14.0) |
| CIT (h) | 8.1 (5.8, 10.8) |
| UW (vs. other) | 297 (74.4) |
|  |  |
| Recipient Characteristic |  |
| Age (y) | 49.5 ± 10.4 |
| Male | 329 (82.4) |
| BMI (kg/m^2^) | 23.0 ± 3.2 |
| MELD score | 23.0 (12.0, 32.0) |
| HCC | 152 (38.1) |
| Acute liver failure ^a^ | 107 (26.8) |
| HBsAg positive | 331 (83.0) |
| Hepatic encephalopathy | 95 (23.8) |
| Hepatorenal syndrome | 45 (11.3) |
| Gastrointestinal bleeding | 34 (8.5) |
| Moderate ascites | 94 (23.6) |
| Waiting time (d) | 19.0 (7.0, 40.0) |
| GWIT (min) | 51.0 (44.0, 58.0) |
| Anhepatic time (min) | 72.0 (62.0, 84.0) |
| Operation time (h) | 5.1 (4.6, 5.9) |
| Immunosuppressant |  |
| IL2R antibody | 343 (86.0) |
| Steroid-free | 216 (54.1) |

^a^: including acute-on-chronic liver failure;

BMI, body mass index; DCD, donation after circulatory death; CVA, Cerebrovascular accident; HBsAg, hepatitis B surface antigen; DWIT, donor warm ischemia time; DHT, donor hepatectomy time; CIT, cold ischemia time; UW, University of Wisconsin; MELD, model for end-stage liver disease; HCC, hepatocellular carcinoma; GWIT, graft warm ischemia time;

Table S2 The potential risk factors of early allograft dysfunction (except the time intervals)

|  | Univariate | | Multivariate ^a^ | |
| --- | --- | --- | --- | --- |
|  | OR (95%CI) | P | OR (95%CI) | P |
| Quantitative data |  |  |  |  |
| Donor Scr | 1.002 (1.000, 1.004) | 0.028 |  |  |
| Donor ALT | 0.997 (0.993, 1.001) | 0.091 |  |  |
| Graft weight | 1.002 (1.001, 1.003) | < 0.001 |  |  |
| CIT | 1.146 (1.069, 1.228) | < 0.001 |  |  |
| Anhepatic time | 1.015 (1.003, 1.028) | 0.012 |  |  |
| MELD score | 1.034 (1.014, 1.054) | 0.001 |  |  |
| Categorical data ^b^ |  |  |  |  |
| Macrovesicular steatosis > 20% | 2.144 (0.885, 5.193) | 0.091 |  |  |
| ABO incompatible | 1.644 (0.953, 2.835) | 0.074 |  |  |
| DCD (vs. DBD) | 1.444 (1.025, 2.034) | 0.036 |  |  |
| Donor Scr > 1.5 mg/dl | 1.762 (1.092, 2.842) | 0.020 |  |  |
| Graft weight > 1.5 kg | 2.788 (1.724, 4.509) | < 0.001 | 2.874 (1.726, 4.788) | < 0.001 |
| CIT > 10 h | 2.447 (1.533, 3.908) | < 0.001 | 1.983 (1.183, 3.326) | 0.009 |
| Anhepatic time > 80 min | 1.686 (1.041, 2.730) | 0.034 |  |  |
| MELD score > 25 | 2.370 (1.525, 3.682) | < 0.001 | 2.036 (1.247, 3.322) | 0.004 |

^a^: only categorical data showing significance in univariate analysis were entered into multivariate analysis. ^b^: cutoff values were selected according to ROC curve considered both sensitivity and specificity. Scr, serum creatinine; ALT, alanine aminotransferase; CIT, cold ischemia time; DCD, donation after circulatory death; DBD, donation after brain death; MELD, model for end-stage liver disease.

Table S3 Graft characteristics in metabolomic analysis

|  | Control group  (n= 43) | EAD group  (n= 24) | PNF group  (n= 14) |
| --- | --- | --- | --- |
| Age (y) | 37.7±16.0 | 41.8 ± 11.6 | 36.0 ± 16.9 |
| Male (n) | 36 | 20 | 11 |
| BMI (kg/m^2^) | 22.6 ± 2.9 | 23.4 ± 3.6 | 22.7 ± 3.2 |
| DCD (n/%) | 30 | 18 | 11 |
| Cause of death |  |  |  |
| Trauma | 25 | 13 | 10 |
| CVA | 15 | 10 | 4 |
| Other | 3 | 1 | 0 |
| HBsAg positive (n/%) | 39 | 20 | 11 |
| Graft weight (kg)^a^ | 1.32 ± 0.26 | 1.53 ± 0.39 | 1.51 ± 0.39 |
| Macrovesicular steatosis > 20% (n/%) | 2 | 3 | 2 |
| DWIT (min) | 10.0 (5.0, 14.0) | 11.0 (8.0, 14.0) | 11.0 (7.3, 19.0) |
| CIT (h) ^b^ | 7.4 (6.0, 10.7) | 9.0 (7.4, 12.5) | 11.3 (9.8, 12.9) |
| Anhepatic time (min) | 69.0 (61.0, 82.0) | 67.0 (60.0, 74.5) | 83.0 (64.5, 101.2) |
| UW (vs. other) | 33 | 14 | 11 |
| Donor TB (mg/dl) | 0.86 (0.61, 1.42) | 0.88 (0.57, 1.12) | 1.17 (0.74, 1.94) |
| Donor ALT (U/L) | 34.4 (23.0, 51.0) | 30.0 (22.0, 48.5) | 49.5 (28.2, 85.3) |
| Donor AST (U/L) | 73.0 (39.0, 116.0) | 44.0 (32.0, 107.5) | 74.6 (44.5, 110.2) |

^a^: EAD vs. control, *P* < 0.05; ^b^: PNF vs. control, *P* < 0.05;

BMI, body mass index; DCD, donation after circulatory death; CVA, Cerebrovascular accident; HBsAg, hepatitis B surface antigen; DWIT, donor warm ischemia time; CIT, cold ischemia time; UW, University of Wisconsin; TB, total bilirubin; ALT, alanine aminotransferase; AST, aspartate aminotransferase;
